# A Review of Per- and Polyfluorinated Alkyl Substance Impairment of Reproduction

**DOI:** 10.3389/ftox.2021.732436

**Published:** 2021-11-22

**Authors:** Weston S. Chambers, Jaida G. Hopkins, Sean M. Richards

**Affiliations:** ^1^ Department of Biology, Geology and Environmental Sciences, University of Tennessee at Chattanooga, Chattanooga, TN, United States; ^2^ Department of Obstetrics and Gynecology, Section on Maternal-Fetal Medicine, University of Tennessee College of Medicine, Chattanooga, TN, United States

**Keywords:** PFAS, reproduction impairment, short-chain PFAS, GenX, ADONA, F53B

## Abstract

In this review article, we compiled peer-reviewed literature describing PFAS exposure and reproductive effects in animals and humans. The aim was to compare environmental occurrence and effects of the most prominent long-chain PFAS compounds and their short-chain replacements. Long-chain PFAS compounds are known to persist in the environment due to their chemical stability, and also known to bioaccumulate; hence, these compounds are being replaced globally. Indeed, PFOA and PFOS are considered long-chain “forever pollutants,” and thus the potential reproductive risk may continue for decades. Much less is known about their short-chain replacements despite the fact that they becoming more widespread in the environment. Short-chain PFAS are generally less bioaccumulative than long-chain, but they are more mobile and persistent in aquatic ecosystems. The three most prominent of these are commonly referred to as GenX, ADONA and F53B. The short-chain PFAS have similar physical and chemical properties as their predecessors; however, because they are relatively new, much less is known about the potential to disrupt reproduction. Indeed, high-quality epidemiological studies are needed to determine associations between short-chain PFAS exposure and effects on reproductive health. However, epidemiological evidence is mounting that long-chain PFAS exposure is associated with reproductive effects (i.e., decrease in fertility, reduced fetal growth and birth weight, pregnancy-induced hypertension and preeclampsia, thyroid hormone disruption during pregnancy, and preterm birth). Evidence from animal models and human cell lines indicates that short-chain PFAS similarly affect reproductive endpoints; however, epidemiological studies are scarce and inconsistent. Although short-chain PFAS have been quantified in drinking water and sediment worldwide, most of these studies did not focus on quantitation of GenX, ADONA, and F53B. There are also many other short-chain PFAS byproducts of manufacturing that have yet to be identified and studied. When sum total concentration of long- and short-chain PFAS are considered, the concentration rises by an order or magnitude or greater, as will the risk of exposure and subsequent reproductive effects.

## Introduction

### Long-Chain PFAS

Per- and Polyfluorinated Alkyl Substances (PFAS) are synthetic chemicals that contain at least one perfluoroalkyl group (Birru et al., 2021). Perfluorooctanoic acid (PFOA) and perfluorooctane sulfonate (PFOS) are the most common PFAS detected in the environment due to their widespread use in manufacturing and chemical stability (Huang and Jaffé, 2019). Both PFOS and PFOA are considered long-chain PFAS because they possess an eight-carbon backbone; their functional groups are sulfonate and carboxylate, respectively (Tsuda, 2016). They possess both hydrophobic and oleophobic properties, along with other chemical characteristics making them useful in many consumer goods (Birru et al., 2021). Their carbon-fluorine bonds, make them resistant to degradation and allow them to persist in the environment and bioaccumulate within living organisms (Zeng et al., 2019; Neagu et al., 2021).

After more than 50 years of production, PFOS and PFOA were widely discovered in humans and the environment, and a subsequent phasing out of production by the 3M company began in 2000 (Giesy and Kannan, 2001; Olsen et al., 2017). Subsequently, the United States Environmental Protection Agency procured an agreement with other major manufacturing companies to reduce long-chain PFAS emissions; other global environmental regulations for PFAS emerged as well (Olsen et al., 2017). The phasing out of these compounds resulted in a decline in human blood serum concentrations of PFOS and PFOA between the years of 2000 and 2015 (Olsen et al., 2017; Wang et al., 2017). Despite the reduction in production of long-chain PFAS, the compounds persist in the environment and in humans and are a continuing concern due to their widespread environmental persistence, distribution, potential toxicological effects, potential to bioaccumulate (Huang and Jaffé, 2019).

Ingestion is considered the largest exposure route to humans (rather than dermal and inhalation). Tap water is a major PFAS exposure route where most average PFAS concentrations are in the low ng/L range (Sinclair et al., 2020). Regarding PFOA and PFOS, the USEPA has issued a Health Advisory Level of 70 ppt and is in the process of making a final regulatory determination (USEPA, 2021). Seafood is a significant PFAS exposure route while exposure from consumer goods, food packaging, and indoor environments are uncertain (Sunderland et al., 2019).

### Short-Chain PFAS Replacements

With the phasing out of long-chain PFAS in the early 2000s, short-chain alternatives (i.e., those with carbon backbones of <7 carbons) began to take their place in industry and the environment (Brendel et al., 2018). These alternatives share similar structures to long-chain PFAS (i.e., heavily fluorinated carbon chains), however, one or more alkylether group is inserted into a shorter fluoroalkyl chain. Short-chain PFAS are also products of long-chain degradation (Li et al., 2020). Short-chain replacements GenX, ADONA, and F53B are widely used. GenX is a trade name for a short-chain PFAS processing technology, of which the hexafluoropropylene oxide (HFPO) dimer acid and its ammonium salt are the major constituents. ADONA (dodecafluoro-3H-4,8-dioxanonanoate) is generally used as a substitute for PFOA in fluoropolymer production (Fromme et al., 2017; Munoz et al., 2019). Chlorinated polyfluoroalkyl ether sulfonate, or F53B, has been produced as an alternative to PFOS and has been adopted as a mist suppressant by several electroplating companies (Du et al., 2016; Munoz et al., 2019; Shi et al., 2019).

## Environmental Concentrations

### Long-Chain PFAS

Water is the major environmental sink for long- and short-chain PFAS. Bai and Son (2021) quantified concentrations of 17 PFAS in the Las Vegas and Reno watersheds (Nevada, USA), and compared them to data on aquatic PFAS concentrations worldwide. Overall, Bai and Son (2021) found that concentrations of individual PFAS ranged from non-detectable to low ppt (maximum of 74.47 ng/L) which was similar to studies and findings in watersheds of the United States (Nakayama et al., 2007; Zhang et al., 2016), Europe (Moller et al., 2010), Uganda (Dalahmeh et al., 2018), China (So et al., 2007; Zhao et al., 2013; Wang et al., 2019), and Australia (Clara et al., 2009). In surface waters where sewage effluent is the primary input, maximum PFAS concentrations were 207.59 ng/L; groundwater contaminated with landfill leachate maximum PFAS concentrations were 5,200 ng/L (Zhou W. et al., 2017). PFAS have been detected in the tissues of many wild species, including, but not limited to, bald eagles, albatrosses, polar bears, seals, dolphins, alligators, squid, and many fish species (Giesy and Kannan, 2001; McCarthy et al., 2017).

### Short-Chain PFAS

Although atmospheric deposition is a possibility, the main source of GenX and ADONA in surface water is manufacturer’s wastewater (Hopkins et al., 2018). F53B is primarily released into waterways via chrome plating industries, finding its way into nearby irrigation systems and ground water. The shorter length and alkylether substitution of short-chain PFAS contribute to a greater water solubility. GenX, when in water, loses an ammonium group, creating an anion that is the same as the HFPO-DA anion in water (Hopkins et al., 2018). This anion has a precursor that travels via air, the C3 dimer acid fluoride (Hopkins et al., 2018). Once the C3 dimer acid fluoride interacts with water, it creates the same anion which readily migrates to groundwater, contributing to private well contamination (Hopkins et al., 2018). The limited data indicate that most short-chain PFAS are more persistent and widespread than long-chain PFAS in the aquatic environment (Olsen et al., 2017; Wang et al., 2017; Brendel et al., 2018; Li et al., 2020). GenX chemicals are present in multiple aquatic environments including surface water, groundwater, and in drinking water and rainwater (USEPA, 2018). Aside from factory wastewater effluent or points very close to industry outfalls, concentrations of GenX, ADONA, and F53B are found in surface waters in the low ng/L concentrations (Pan et al., 2018; Munoz et al., 2019; Gebbink and van Leeuwen, 2020; Bai and Son, 2021). Both GenX and F53B were found in higher concentrations than long-chain PFAS in surface water samples collected downstream from a PFAS manufacturer (Gebbink et al., 2017; Ateia et al., 2019). And although short-chain PFAS have been quantified in drinking water and sediment worldwide, most studies did not focus on quantitation of GenX, ADONA, and F53B and thus, data in the aquatic environment are lacking (Ateia et al., 2019).

Bai and Son (2021) found that short-chain PFAS were more likely to partition in the water column, while long-chain PFAS most likely to partition in sediment. This is similar to the finding of Zhao et al. (2016) who estimated that 88.8% of total PFAS in the water column are short-chain. These estimations correspond to what would be expected based on the greater water solubility of short-chain PFAS.

It is important to note that the studies that concluded “relatively low” concentrations of PFAS in the water column or sediment are often describing the concentration of *individual* molecules. While environmental regulations limit concentrations of individual PFAS, the sum of the thousands of PFAS need to be considered for estimations of exposure and effects. Indeed, even if toxicity is considered to be simply additive, estimations of risk must include total PFAS exposure.

## PFAS Presence in Humans

Routes of human exposure to PFAS include food, drinking water, house dust, ambient and indoor air, and consumer products (Olsen et al., 2017). Examples of consumer products that have utilized PFAS in the past include fabrics, carpet, grease-proof food-contact paper, nonstick cookware, paints, and cosmetics (Kjeldsen and Bonefeld-Jørgensen, 2013; Olsen et al., 2017).

For populations close to sources of contaminated water sources, drinking water is the dominant exposure pathway (Vestergren and Cousins, 2009). This was illustrated by Fromme et al. (2017); blood plasma samples were tested for the presence of PFOS, PFOA, and ADONA in humans living within an 80 km radius of a former PFOA production plant, where ADONA was being used as a replacement emulsifier. Drinking water was the known exposure route. The 95th percentile concentrations in blood plasma were 13.5 μg/L and 85.5 μg/L for PFOS and PFOA, respectively. Blood plasma concentrations of ADONA were just above detection levels (0.2 μg/L), indicating that health risks were not likely (Fromme et al., 2017).

Food is also a major source of exposure, PFAS have been detected in fish fillets, blood serum of beef cattle, fruits and vegetables, bread, and milk (Vestergren and Cousins, 2009). Human breast milk is a source of infant exposure to PFAS, and these compounds have been detected in samples in the United States, Asia, and Europe (Tao et al., 2008). PFAS have also been found in human umbilical cord blood samples, with one study detecting trace amounts in 100% of tested samples (Apelberg et al., 2007). These findings, indicating an exposure pathway *in utero*, are concerning due to the high vulnerability to toxicants during fetal development (Bach et al., 2014).

PFOS and PFOA are readily absorbed in the gastrointestinal tract, excreted in urine and feces, and do not undergo metabolism. Estimated human half-lives for PFOS and PFOA are approximately 5 years and 2–4 years, respectively (EFSA, 2018). PFAS bind to blood serum proteins, allowing them to travel through the circulatory system and subsequently accumulate in most organs in the human body, particularly in the kidneys, liver, spleen, brain, and testicles (Jensen and Leffers, 2008; Wang et al., 2021). Elimination of PFAS from the body is done via renal clearance. Clearance varies widely by species. In humans it is a relatively slow process with half-lives of 3.8 years for PFOA and 5.4 years for PFOS (Behr et al., 2018). Much shorter half-lives are observed in other animals, with male mice, rabbits, chickens, and cynomolgus monkeys having half-lives of 12, 0.23, 4.6, and 30 days, respectively (Vestergren and Cousins, 2009). For water-breathing organisms, the high aqueous solubility allows for easier elimination of the compounds through the gills (Kelly et al., 2009).

## Bioaccumulation

Another reason for the phasing out of long-chain PFAS is the potential of these compounds to bioaccumulate (Olsen et al., 2017). In Lake Ontario, the concentrations of long-chain PFAS were higher within the biota and sediment than the water, giving a bioaccumulation factor between lake trout and water of 3.4 × 10^4^ L/kg (Houde et al., 2008). Zooplankton contained 195 ng/g, sediment contained 187 ng/g, and the water contained 2.2 ng/L (Houde et al., 2008). Some predatory species have been observed to contain higher levels of PFAS than in their diets, indicating that PFAS can biomagnify in higher trophic levels (Giesy and Kannan, 2001). Biomagnification is seen only in non-aquatic predators, such as seals and polar bears, most likely due to the proteinophilic nature of PFAS, relatively high aqueous solubility of PFAS, and the low volatility (Kelly et al., 2009).

Because of the increased water solubility of short-chain PFAS, most studies indicated a lower bioaccumulation potential. Indeed, short-chain PFAS are eliminated from the body quicker, and therefore have less potential to bioaccumulate (Gomis et al., 2018). Carbon-chain length is generally inversely proportional with elimination rates; this trend follows for humans as well (Lau, 2012). For example, a four carbon PFAS, perfluorobutanoate (PFBA), had a measured half-life of 3 days in humans, which is a stark difference from the years-long half-lives of PFOS and PFOA (Lau, 2012). While short-chain PFAS may not remain in biota for extended periods of time, this does not necessarily indicate that they have less potential toxicity than long-chain PFAS (Gomis et al., 2018). Short-chain PFAS are still very persistent in the environment, indicating that there is potential for high environmental exposure (Gomis et al., 2018). However, the faster rate of clearance of short-chain PFAS could potentially reduce developmental toxicity (Lau, 2012). Also, there is no current evidence that biotransformation of these chemicals takes place within the body due to their structural stability, however, precursors to these molecules have exhibited biotransformation into perfluoroalkyl acids (Sunderland et al., 2019).

While it is generally accepted that short-chain PFAS bioaccumulate less than long-chain PFAS, the bioaccumulation of ADONA and GenX is not well researched (Brendel et al., 2018; Munoz et al., 2019; Fan et al., 2020). To further complicate the issue of short-chain PFAS bioaccumulation, there is some evidence that F53B may biomagnify in aquatic food webs (Munoz et al., 2019). To address this data gap, integrative computational approaches are being used to estimate PFAS bioaccumulation effects across several species. This approach found a pattern of similar binding affinities to liver fatty acid-binding protein across nine different species, including humans, chickens, and rainbow trout (Cheng et al., 2021). This approach indicates similar PFAS bioaccumulation potentials across species and illustrates how computational approaches can be used to estimate binding to other receptors and ligands. However, the bioaccumulation potential of short-chain PFAS needs to be further researched in live animal models.

## PFAS Reproductive Effects

### Long-Chain Reproductive Effects in Animal Models

Researchers have utilized many different model organisms in order to better understand the reproductive effects of PFOS on living organisms. Shi et al. (2008) observed zebrafish embryos exposed 4 h post-fertilization to varying concentrations of PFOS between 0.1 and 5 mg/L, and observed significant changes in embryo development. Noted changes included delayed hatching, malformation, and depressed heart rates, which led to reduced survival rates of fry exposed to 1 mg/L PFOS for 21 days before hatching (Shi et al., 2008). Han and Fang conducted a study to observe the effects of PFOS exposure on swordtail fish; they found a significant decrease in survival rate of offspring at higher levels of PFOS exposure in these swordtail fish (Han and Fang, 2010). PFOS exposure has been associated with inhibition of sperm production (Qu et al., 2016). In female mice, PFOS exposure is associated with ovulation reduction (Wang et al., 2018). Exposure of pregnant mice to PFOS resulted in neonatal mortality as a result of severe lung collapse and intracranial blood vessel dilation (Yahia et al., 2010).

While PFOA is similar to PFOS in structure, PFOS is generally considered to be more toxic (Zheng et al., 2012). Similar research has been conducted on PFOA as that on PFOS in order to understand the reproductive effects of the compound. A study was conducted to examine the effects of PFOA on development of zebrafish and found that PFOA is acutely toxic to zebrafish embryos (LC_50_ = 262 mg/L), similar to PFOS (Zheng et al., 2012). At higher concentrations, edema, delayed hatching, and spinal malformations were observed, and nearly 100% mortality was observed when the embryos were exposed to 270 mg/L (Zheng et al., 2012). A meta-analysis was conducted to determine the effects of PFOA on the reproductive system of male rats and found significant association between higher PFOA levels and reproductive toxicity (Wang et al., 2021). Toxicological effects of increased serum PFOA levels included a decrease in serum testosterone levels and a decrease in weight of reproductive organs (testicle and epididymis) (Wang et al., 2021). The exposure of PFOA to pregnant mice resulted in reduced fetal body weight and delayed bone formation, and a 100% mortality rate of pups after exposure at 10 mg/kg was observed (Yahia et al., 2010). PFOA increased mortality of the pups, similarly to PFOS, but no intracranial blood vessel dilation was observed, indicating a different cause of death by PFOA (Yahia et al., 2010).

PFAS interact directly with estrogen receptor α (ERα) in trout liver *in vivo*, although they have a weak affinity and they are noted to induce an estrogenic effect (Benninghoff et al., 2010). In male mice, PFOS exposure was observed to down-regulate ERα and up-regulate estrogen receptor β, which increased cell apoptosis and decreased cell proliferation in the testes (Qu et al., 2016). In female mice, PFOS was shown to suppress ERα receptors, which led to induced prolongation of diestrus and ovulation reduction (Wang et al., 2018). Accordingly, Jensen and Leffers (2008) found that male rats exposed to PFOA developed Leydig cell hyperplasia eventually resulting in adenomas (Jensen and Leffers, 2008). A decrease in testosterone production was also reported in these rats followed by an increase in estradiol levels.

Reproductive functions are dependent on peroxisome proliferator-activated receptors (PPARs). These receptors are susceptible to PFAS which are known to affect gene transcription through the activation of PPARs (Vitti et al., 2016). When mice were treated with PFOA, subsequent changes in PPARs were associated with delayed mammary gland development (Zhoa et al., 2012).

### Short-Chain Reproductive Effects in Animal Models

Three recent studies examined reproductive effects of GenX in mice (Blake et al., 2020) and rats (Conley et al., 2019; Conley et al., 2021). Mice treated with GenX had a greater incidence of placental abnormalities while rats showed a greater incidence of reduced maternal thyroid hormone levels and elevated levels of PPAR-regulated gene expression in both maternal and fetal livers (as with PFOA described above). GenX is a developmental toxicant in the rat; those dosed from gestational day 8 through post-natal day 2 (from 1 to 125 mg/kg/day), had increased neonatal mortality and reduced birth weight (Conley et al., 2021).

In zebra fish, F53B was associated with an increase in birth defects, delayed hatchings, and decreased survival rates in embryos (Shi et al., 2017). Conversely, Gaballah et al. (2020) concluded that GenX (and ADONA) was not associated with developmental toxicity in zebrafish.

### Long-Chain Reproductive Effects in Human Cell Lines

There is conflicting evidence regarding whether or not PFAS interact with estrogen receptors in humans. Multiple studies have concluded that PFAS promote activity of estrogen receptors in the presence of estradiol, but do not directly interact with them (Kang et al., 2016; Behr et al., 2018). Another study found that PFAS do not have any effect on ER, while another found that PFAS bind directly to ER without the presence of estradiol (Kjeldsen and Bonefeld-Jørgensen, 2013; Yao et al., 2014). Despite being heavily researched, a lack of understanding of the mode of action of these pollutants persists (Cousins et al., 2020).

Zhang et al. (2015) exposed human syncytiotrophoblasts isolated from term placenta to PFOS. Dose-dependent reductions of human chorionic gonadotropin (hCG), estradiol, and progesterone resulted. After blocking the apoptotic route, the authors concluded that PFOS exerted its effects by inducing apoptosis due (Zhang et al., 2015).

## Epidemiological Studies of PFAS

### Long-Chain PFAS

Multiple epidemiological studies have been conducted to determine the associated effects of PFAS on humans. Johnson et al. (2014) found an association between lower birth weights and exposure to PFOA, noting an 18.9 g decrease in birth weight for every ng/mL increase in serum PFOA level. As described in rat models above (Jensen and Leffers, 2008), Leydig cell hyperplasia is connected with lower testosterone levels in men, indicating a possible mode of action for PFOA to induce testicular cancer (Tarapore and Ouyang, 2021).

As also described above, the effects of PFAS on human reproductive hormones is uncertain. One epidemiological study found that an increase in human serum PFAS levels was negatively associated with serum levels of reproductive hormones, including testosterone (Tsai et al., 2015). However, another study found a positive correlation between increased PFAS levels and levels of testosterone and estradiol, while yet another study found no correlation between PFAS and sex hormone levels (Olsen et al., 1998; Sakr et al., 2007). PFAS have also been shown to compete with thyroxine to bind to transthyretin (Kar et al., 2017). Both molecules play a major role in thyroid function (Kar et al., 2017). Luo et al. (2021) recruited 902 men for a cross-sectional study of 24 PFAS compounds in blood plasma. Five reproductive hormones: total testosterone (TT), E_2_, FSH, LH, and insulin like factor 3 (INSL3), and SHBG were measured. They found a significant inverse relationship between PFAS mixture with E_2_ and E_2_/TT. Xie et al. (2021) studied a large cohort of males and females aged from 12 to 80 and found that PFAS exposure was associated with alterations in sex hormones (including TT, free testosterone, E_2_, and SHBG) in a sex-, age-, and compound-specific manner. Multiple epidemiologic studies (Taylor et al., 2014; Barrett et al., 2015; Zhou Y. et al., 2017) associate PFAS exposure with effects indicating ovarian alterations (e.g., altered menstrual cycle, hormone alterations, early menopause). Petersen et al. (2018) examined PCBs and PFAS blood concentrations and associated effects on semen quality and reproductive hormones in Faroese men. Concentrations of total PCBs and PFOS were positively associated with sex hormone-binding globulin (SHBG) and luteinizing hormone (LH). While the PCB concentrations in the Faroese men were relatively high (compared to men around the world), the PFAS concentrations were relatively similar. Overall, the authors concluded that the positive association to LH for both PCBs and PFOS indicate a direct toxic effect on Leydig cells.

Petersen et al. (2020) qualitatively assessed 26 epidemiological studies for evidence of associations between PFAS exposures and male reproductive health. Several studies found some evidence that single PFAS compounds were associated with specific male reproductive parameters (e.g., semen quality, reproductive hormones, cryptorchidism, hypospadias, and testicular cancer). However, the overall conclusions were limited due to a lack of consistency between studies.

### Short-Chain PFAS

Data for short-chain PFAS effects on human reproductive parameters are scarce and these data are inconsistent (Nian et al., 2020; Brase et al., 2021). Indeed, the threat that these long-chain replacements pose on human populations, pathophysiology and health effects are not completely understood (Sunderland et al., 2019). Effects in humans are difficult to extrapolate from laboratory dose-response results. However, existing data indicates that concentrations in humans are lower than dose-response effects found in the laboratory. For example, So et al. (2007) found a maximum human PFAS concentration of 360 ng/L (breast milk), orders of magnitude below laboratory animal model exposures which induced toxicity.

Because of the likelihood of co-occurrence, short- and long-chain PFAS have been studied together. A three year study of 752 women in China found prenatal exposure to one short-chain and one long-chain PFAS (perfluorobutanesulfonate, PFBS and perfluoroheptanoic acid, PFHpA, respectively) was associated with alterations of fetal gonadotropins as well as free androgen levels (Nian et al., 2020). A recent review of endocrine disruption effects associated with both long- and short-chain PFAS exposure found that effects varied similarly according to gender and age of development; in some cases, short-chain effects were greater than long-chain PFAS (Mokra, 2021). While much more is needed pertaining to human reproductive effects, the implications of these studies warrant further epidemiological studies on short-chain PFAS.

## Data Gaps and Limitations

There are similar data gaps and limitations between wildlife and human PFAS exposure reproductive effects. Statistically representative population surveys are lacking for assessing PFAS exposure in wildlife (DeSilva et al., 2021). Laboratory, field, and epidemiological studies are needed to effectively assess effects and risk of PFAS compounds in singular exposure and mixtures. However, because almost 20 years of data exist for long-chain PFAS, there is a considerably greater dearth of data on next-generation PFAS as well as next-gen precursors and degradation products. Mixture toxicity data for all PFAS are woefully inadequate which further complicates our understanding of potential sub-lethal and chronic effects. Molecular markers or metabolic fingerprints for PFAS exposure and effects are needed to effectively develop effective PFAS regulations (Sinclair et al., 2020). Petersen et al. (2020) cite a glaring knowledge gap regarding exposures prior to adulthood, exposure to mixtures of PFAS compounds and other potential endocrine disruptors. Epidemiological studies conducted to date are limited in the conclusions and comparisons that can be made because of inconsistency between studies. High-quality epidemiological studies are needed to determine associations between PFAS exposure and effects on male reproductive health (Petersen et al., 2020).

Data on the effects of PFAS at environmentally-relevant concentrations are limited. This hampers the effective management of these compounds.

There is some evidence that toxicity detected in laboratory animal models and human cell lines occurs at higher PFAS concentrations than what occurs in the environment. While there is some evidence to support this from the HQ perspective, there are significant limitations to the HQ approach. Because the HQ method is based on LC50 values, it tends to neglect the subtle non-lethal effects associated with reproduction. In addition, the dramatically different half-lives described herein further complicates blanket statements about PFAS risk across multiple species. Indeed, the HQ approach could underestimate non-lethal effects and subsequent effects in reproduction of wildlife and humans alike.

Despite HQ values <1, many epidemiological assessments of PFAS exposure indicate correlations with non-lethal reproductive effects. The USEPA cited concerns about effects on reproductive parameter in their recent drinking water health advisory for PFOA and PFOS (USEPA, 2021). Given the similarities of short-chain PFAS to these compounds, concern for effects at environmentally-relevant concentrations is warranted.

Phospholipids and proteins affect tissue partitioning and accumulation of PFAS differently than other long-lived, bioaccumulative toxicants. More research on PFAS mode of action is needed (e.g., endocrine disruption) as well as differences in PFAS toxicokinetic properties between compounds. Indeed, the unique toxicokinetics of PFAS, make standard bioaccumulation metrics unsuitable. Improved PFAS bioaccumulation models are being developed which should lead to more accurate risk assessments in wildlife and humans (DeSilva et al., 2021). This is imperative to understand differing responses and half-lives between the sexes and among species and life stages (Fenton et al., 2021).

Most PFAS research has been conducted on but a few prominent compounds with numerous associated reproductive health impairments. There are thousands of PFAS compounds cycling through the environment and industry for which toxicity data are non-existent. To adequately address this enormous issue more contemporary and high-throughput approaches such as read-across, molecular dynamics, and protein modeling are needed (Fenton et al., 2021).

Improvements are needed in human modelling of exposure, focusing on PFAS exposure susceptibility windows and statistical modeling of data to account for endocrine disruption (Tarapore and Ouyang, 2021). However, care must be taken to address mixture issues, and properly attribute toxic effects and mechanisms to a specific PFAS or mixture of PFAS.

To properly estimate exposure potential, data on environmental concentrations need to be integrated to develop mass balance models of PFAS in different matrices (e.g., land, water, air), hydrologic sub-divisions, and to track the migration pathway of short-chain PFAS to drinking water (Ateia et al., 2017). Short-chain PFAS are resistant to conventional municipal surface water purification methods (Ateia et al., 2019). While drinking water purification trials using granular activated carbon have reported a removal efficiency of 99% (Du et al., 2016), large-scale, affordable municipal drinking water methods are not widely available.

## Conclusion

The chemical properties of PFAS are valuable for manufacturing and consumer convenience; however, these same properties make them resistant to degradation and persistent in the environment, wildlife, and humans. Generally, longer carbon chains in PFAS correlate with higher toxicity, however, differential reproductive effects are observed *in vitro* and *in vivo*. Short-chain PFAS tend to bioaccumulate less, but are more persistent in surface water. While surface water data show *individual* short-chain PFAS presence to be in the low ng/L range, sum *total* concentrations of PFAS are orders of magnitude higher. Some studies indicate that reproductive effects of PFAS seen in laboratory models and human cell lines are at concentrations greater than what will be realized in the environment. Most studies do not consider mixture toxicity; epidemiological studies inherently address mixture toxicity. Epidemiological studies indicate that environmentally-relevant PFAS concentrations are associated with reproductive effects. Indeed, even though most ecological and epidemiological studies quantify a few PFAS, these studies are effectively assessing the impact of many more PFAS. The majority of evidence for reproductive effects is linked to long-chain PFAS exposure. Long-chain PFAS is on the decline worldwide but will remain an exposure issue. Short-chain PFAS exposure is on the rise. Even though some specific short-chain PFAS have been studied, data on reproductive effects of short-chain PFAS are considerably less than for long-chain. In addition, there are many short-chain PFAS byproducts from manufacturing that have yet to be identified.
